# Can HRV Biofeedback Improve Short-Term Effort Recovery? Implications for Intermittent Load Sports

**DOI:** 10.1007/s10484-020-09495-8

**Published:** 2021-01-05

**Authors:** Mauro Perez-Gaido, Jaume F. Lalanza, Eva Parrado, Lluis Capdevila

**Affiliations:** 1 Department of Basic Psychology, Universitat Autónoma de Barcelona, Bellaterra, Barcelona, Spain; 2 Department of Psychology, UiT The Arctic University of Norway, Tromsø, Norway; 3 Sport Research Institute UAB, Universitat Autònoma de Barcelona, Bellaterra, Barcelona, Spain

**Keywords:** Recovery, Breathing, Resonance frequency, HRV, Biofeedback, HRVB

## Abstract

As intensity and physical demands continue to rise in sport competition, faster and better recovery becomes essential. The aim of this study was to assess the effects of HRV biofeedback (HRVB) while recovering from a submaximal aerobic exercise. Ten physically-active graduate students participated in the study, which was conducted in four sessions: exercise with free-breathing recovery, first resonance frequency (RF) detection, second RF detection, and exercise applying HRVB during recovery. Measurements included time spent running and recovering, HRV parameters, and recovery/exertion perceptions. The results indicate that using HRVB during recovery improves cardiac variability (RRmean, SDNN, RMSSD and LF; *p* < 0.01). HRVB also lowers recovery time (*p* < 0.05) and seems to be improving the perception of recovery (*p* = 0.087). Moreover, time spent exercising (*p* < 0.01) and perceived physical exertion (*p* < 0.05) were higher when applying HRVB. The improvement in the psychophysiological adaptation after intensive aerobic exercise provided by the HRVB is a valuable benefit, not only for competition-driven athletes, but also for the general population.

In modern sport competition, intensity and physical demands continue to rise, making recovery a precious resource to minimize overtraining, injury occurrence and increase performance. In order to continue performing at maximum level, it becomes paramount to develop and apply strategies that lead athletes to regain an optimal psychophysiological state as fast as possible. Kellman and Kallus (2001) defined recovery as a process that restores the abilities related to performance. It has intra- and inter-individual components, such as psychological, physiological, and social processes. Several strategies has been reported to achieve a better recovery state, including an individual and sport-specific diet (Wardlaw and Smith 2006), proper hydration, passive recovery, sufficient sleep, physical and mental relaxation techniques (Kudlackova et al. 2013), massages (Weerapong et al. 2005), active recovery and stretching (Bahnert et al. 2013). Current methods of recovery assessment include physiological (heart rate variability, HRV; level of lactate on blood; etc.) and psychological measures (self-report questionnaires, e.g. POMS, REST-Q-Sport; Total Recovery Questionnaire, TQR; Kenttä and Hassmén 1998).

HRV parameters are currently widely used as recovery markers (Buchheit 2014; Djaoui et al. 2017; Michael et al. 2017). HRV could be defined as the variation in time intervals (R–R intervals) between adjacent heartbeats (Shaffer and Ginsberg 2017). Higher levels of HRV are associated with health, adaptability, resilience, and self-regulatory capacity (McCraty and Shaffer 2015). HRV parameters reflect regulation of autonomic balance, blood pressure (BP), gas exchange, heart, and vascular tone. Different HRV parameters reflect different phenomena, and they are divided into time domain and frequency domain parameters. Among the time domain parameters, the standard deviation of all R–R intervals (SDNN) reflects all the cyclic components responsible for variability in a given period (Laborde et al. 2017). The root mean square of successive differences (RMSSD) represents the vagal tone, and is more influenced by the parasympathetic nervous system (PNS Hill et al. 2009). Frequency domain parameters mainly include low frequency (LF), high frequency (HF), and the ratio of LF to HF (LF/HF). LF indexes a mix of activity from the sympathetic nervous system (SNS), vagus nerve, and baroreflexes. LF is highly influenced by slow paced breathing (less than 8.5 breaths per min; Sowder et al. 2010). LF/HF represents a mix of SNS and vagal activity, whereas HF power is generated by the PNS. In short-term recordings of SDNN, specifically, the source of its fluctuation is respiratory sinus arrhythmia (RSA; Shaffer et al. 2014). In addition to the time and frequency parameters of the HRV, there are non-linear parameters, such as approximate entropy, which are also widely used (Huikuri et al. 2003).

Continuing on Vaschillo’s original studies in Russia, Lehrer et al. (2000) published a manual for training in resonant frequency biofeedback (currently called HRV biofeedback, HRVB) to increase cardiac variability. Such work was the underpinning of almost two decades worth of research and applied techniques. By stimulating the cardiovascular system at a specific frequency, HRVB produces very large sinusoidal oscillations in heart rate (HR) and in BP, a phenomenon called RSA. This is a resonance effect triggered by breathing at a certain frequency, usually called resonance frequency (RF).

Several applications using HRVB have been discovered over the years. A recent systematic review and meta analysis (Lehrer et al. 2020) focused on biological, psychological and performance-related issues, and found small to moderate effect sizes in HRVB treatments. However, valuable results were found among clinical applications on asthma, chronic obstructive pulmonary disease, functional gastrointestinal disorders, fibromyalgia, hypertension, chronic muscle pain, gynecology, depression, anxiety disorders, and sleep (for a review: Gevirtz 2013). HRVB has been used to measure training status and adaptive physical processes (Buchheit 2014; Nakamura et al. 2017). Moreover, a review by Jiménez and Molina (2017) reported that this technique has shown improvement in performance in areas such as baseball, golf, dance, music, basketball, volleyball, long-distance running, and soccer. Within different sports, HRVB has been used to improve concentration, anxiety, stress, and emotional control (Lagos et al. 2011; Paul and Garg 2012; Paul et al. 2012; Whited et al. 2014; Gross et al. 2016; Rijken et al. 2016). Moreover, in aerobically fit individuals, HRV recovers faster after exercise (Stanley et al. 2013). As indicated by Peçanha et al. (2013), it is necessary to foster interventions that have a positive impact on HRV, which is known to decrease after exercise.

A thorough search of the relevant literature yielded no results regarding any HRVB application for short-term recovery after an effort or physical stress. Improving recovery would be useful for athletes and any person interested in practicing sports or physical exercise. Therefore, the aim of this study was to apply HRVB after making a physical effort to allow a reduction in the time spent to recover and provide a better psychophysiological state for the next bout of exercise. It is hypothesized that participants, by using HRVB during recovery, will have a better psychophysiological adaptation during the recovery-stress process.

## Method

### Participants

Fifteen physically active graduate university students (7 males), ranging from 23 to 35 years (25.80 ± 4.18 years), participated in this study. The level of exercise was the inclusion criteria, and it was measured using the Spanish adapted version of the Physical Activity Stages of Change Questionnaire (PASC-Q; Marcus et al. 1992). This only included participants that exercised a minimum of three times a week, at least 30 min each session, and were at least in action phase (according to the Transtheoretical Model; Marcus and Simkin 1994). All participants that answered positively to any item of the Physical Activity Readiness Questionnaire (PAR-Q, Thomas et al. 1992) or were unable to run past the warm-up phase, were excluded from the study. Of the 15 participants that started the study, 10 were finally considered for data analysis (see Table 1) and 5 were excluded: 1 did not complete all of the sessions, 1 was excluded because of a positive item in PAR-Q, 2 were disregarded for not completing the warm-up phase, and 1 for coughing during a recovery phase. Privacy was assured for all participants as regards all data collected. All participants were volunteers and provided written informed consent. The study was reviewed and approved by the local ethics committee and meets the Ethical Standards in Sport and Exercise Science Research (Harris and Atkinson 2009).

**Table 1:** Descriptive statistics for participants

	Mean	SD
Age	25.80	4.18
Height	174.85	8.69
Women	167.00	3.00
Men	178.21	8.13
Weight	75.54	8.37
Women	64.93	5.40
Men	80.09	3.87
Exercise per week	430.50	199.51
Time exercising	38.90	20.14

*Height* expressed in centimeters, *weight* in kilograms, e*xercise per week* in minutes and *time exercising* in weeks. *n* = 10

### Measures

#### Cardiac Measures

The PolarBand H7 (Polar Electro, Finland) was used to detect the R–R intervals signal. HealthSportLab software (HealthSportLab S.L., Barcelona, Spain) was used to detect and collect the R–R interval signal and calculate the cardiorespiratory variables. For time domain analysis, HR average (HRmean), R–R intervals average (RRmean), SDNN, and RMSSD were calculated, and for frequency domain, low frequency (0.04–015 Hz) and LF/HF ratio were computed. During recovery phases, measurements were made in bouts of 90 s (Ultra Short Term HRV; Esco et al. 2018; Melo et al. 2018; Nakamura et al. 2017). HRV analysis complies with all guidelines recommended by the Task Force of the European Society of Cardiology and the North American Society of Pacing and Electrophysiology (1996) for measurement of HRV.

#### Self-reported Measures of Recovery and Physical Exertion

Perception of recovery was assessed using a modified version of the Total Quality Recovery perceived scale (TQRper; Kenttä and Hassmén 1998). Scores on this version range from 1 to 10, where “1” corresponds to no recovery at all, and “10” to maximal recovery. Perception of exertion was evaluated using the Borg’s Category Ratio Scale (CR-10; Borg and Löllgen 2001). Scores range from 1 to 10, where “1” corresponds to no exertion, and “10” to maximal exertion.

### Procedure

**Fig. 1:**
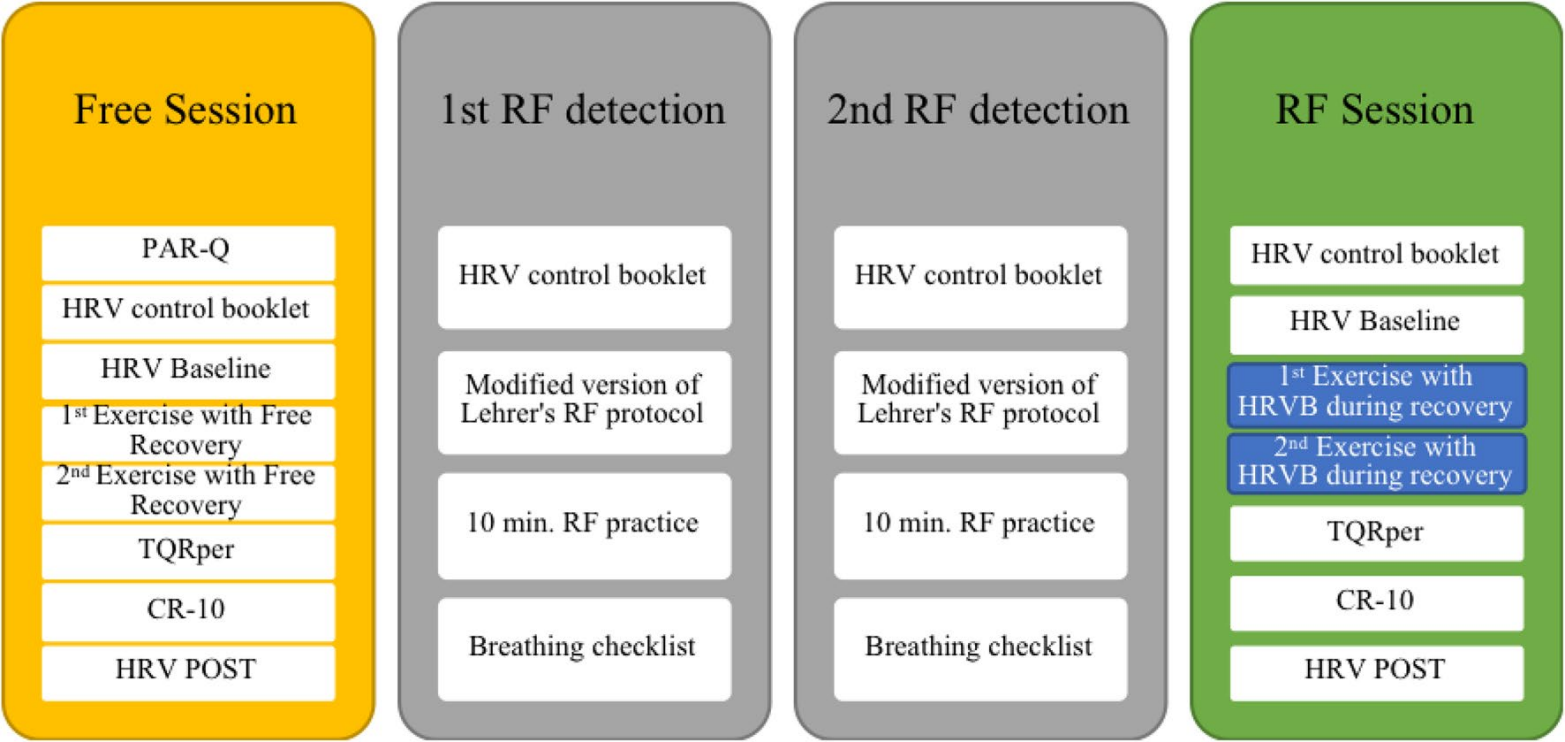
Diagram of the different sessions

A within-subject design was used in this study. Participants were contacted via email. After completing an online version of the PASC-Q, they were selected according to the inclusion criteria. The study was conducted in four sessions, in chronological order (see Fig. 1): (1) exercise with free-breathing recovery (Free Session); (2) first RF detection; (3) second RF detection; and (4) exercise with HRVB recovery (RF Session). The sessions were approximately 1 week separated from each other. Before every session participants completed a HRV control booklet (Parrado et al. 2010) to assess behaviors that could affect HRV measurement (cardioactive medication, physical activity practice, caffeine, tobacco, alcohol, food intake, sleeping quality and quantity, and taking of contraceptives), as recommended by Laborde et al. (2017). Participants were asked to control such behaviors to the maximum of their capability in the hours before to the sessions. In the first session, participants completed an informed consent form and the PAR-Q questionnaire. Sub-maximal HR (80%) was calculated for each participant in order to control their effort during exercise, using Tanaka’s formula (2001): HRmax = 208 − 0.7 × age. This formula was validated in runners Nikolaidis et al. (2018).

**Fig. 2:**
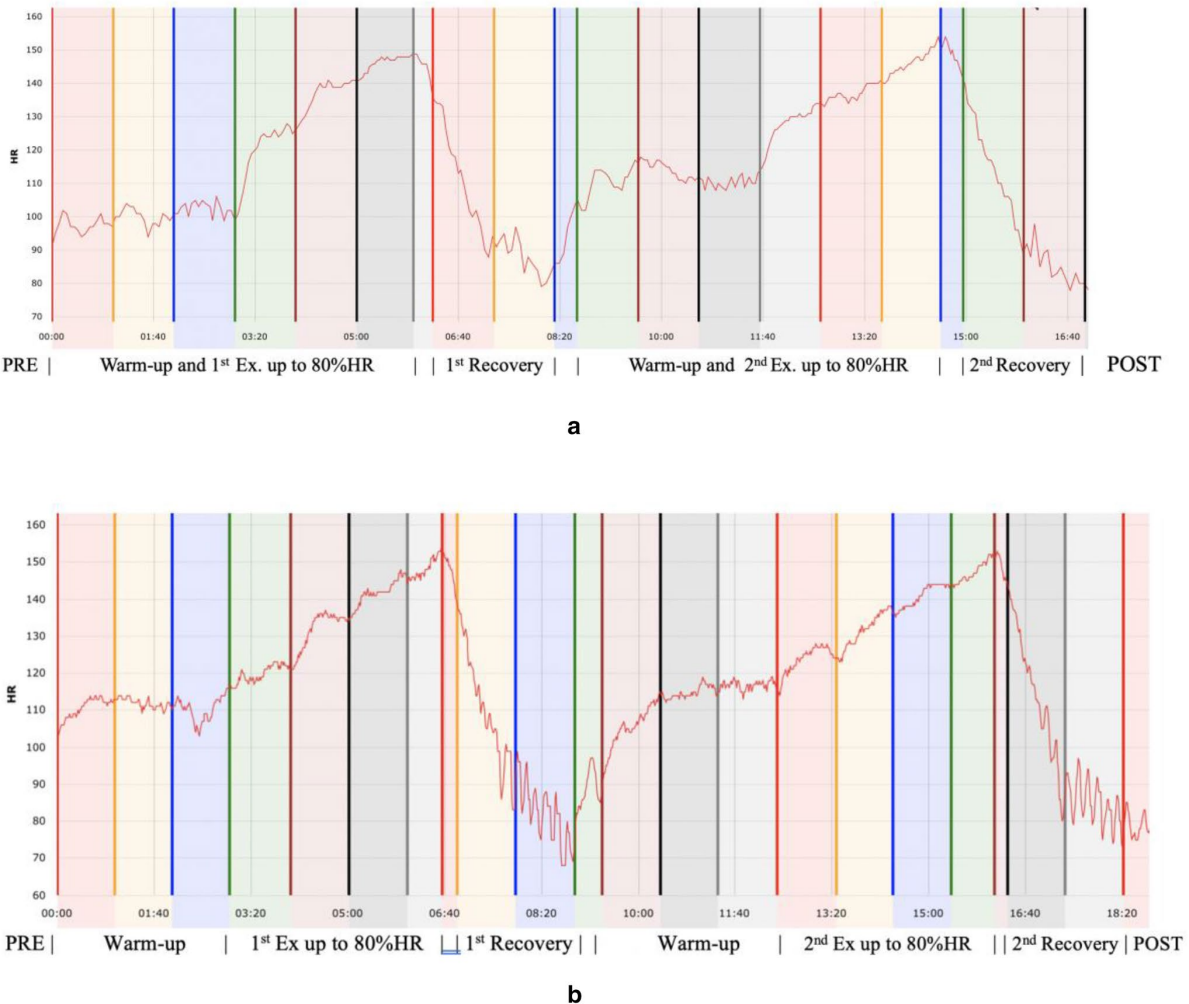
**a** Example of HR plot for a Free Exercise session (Participant 9). Below the graph there are the different phases of the session. *Heart Rate (HR)* expressed in beats per minute; *Time* in X axis expressed in minutes and seconds. **b** Example HR plot of a RF Exercise session (Participant 9). Below the graph there are the different phases of the session. *Heart Rate (HR)* expressed in beats per minute; *Time* in X axis expressed in minutes and seconds

The objective of the first session (Free Session, see Fig. 2a) was to perform and recover twice from a submaximal aerobic exercise test by the participants, without giving any instructions regarding how they should breath during recovery. First of all, a 5-min HRV baseline (PRE) was measured while the participant was seated on a chair. Afterwards, the exercise started with a warm-up, running for 3 min at a speed of 6 km/h and an inclination of 1%. The speed was then increased by1 km/h each minute (e.g., 3rd–4th min 7 km/h, 4th–5th min 8 km/h, etc.). When the participants reached 80% of their HR, the speed of the treadmill was slowly decreased until full stop. The participant was instructed to sit on a chair for 2 min. No breathing instruction was given. Immediately afterwards, the exercise test and subsequent recovery period was repeated according to the same procedure. After the second recovery, the subjective measurements of TQRper and CR-10 were taken using a written scale. Finally, a 5-min HRV (POST) assessment was performed. Exercise performance was calculated as the time spent running from the start of each exercise (including the warm-up) until the end of a 15-s period with at least 80% HR. Recovery time was calculated as the time taken to reach 50% HR, immediately after sitting down on the chair, in each recovery phase.

In the second and third session, participants attended the RF detection sessions to assess their RF and then, learn and practice the individualized breathing protocol. RF was assessed following a modified version of the protocol of Lehrer et al. (2000). The modification consisted of the breathing frequencies used (7, 6.5, 6, 5.5, and 5 breaths per min). Following an auditory pacer, the participants breathed at every frequency for 2 min each without a pause in-between and at the same inspiration/expiration ratio (without holding). The RF was selected automatically by the FitLab Team app (HealthSportLab S.L., Barcelona, Spain), taking into account the highest RSA amplitude during the different frequencies. Afterwards, participants practiced following their RF for 10 min using Breath Pacer app (T.C. Applications Inc., Toronto, Canada), while measuring HRV, and finally, completed a breathing checklist to assess whether it was comfortable to follow the RF.

The last session (RF session, see Fig. 2b) was aimed at using the RF while recovering after the exercise bouts. Taking into account that RF can be not stable, the frequency used in the last session was calculated as the mean between the two frequencies obtained in both RF detection sessions. The mean of the RF used by the participants during the application of HRVB was 5.7 breaths/min (0.6). This session was conducted in the same manner as the first session: first exercise test and first recovery phase; second exercise test and second recovery phase. The difference was in the recovery phases in which the HRVB protocol was applied. The HRVB protocol consisted of instructing the participant to breathe first at double the individual RF for 30 s and then at RF for 90 s (always using the same auditory and visual pacer as the RF detection sessions). The period of 30 s breathing at double the RF was meant to provide a better adaptation to the paced breathing, since RF is low and difficult to follow, after a sub-maximal physical exercise test.

### Statistical analysis

All data were reported as means (standard deviation, SD). The standard error of the mean (SEM) is reported in the figures. The significance level used is *p* < 0.05. A maximum signal error for RR records of 11% was accepted and filtered. The mean of signal error for all RR records was 0.67%. One participant was discarded from the PRE assessment data due to software malfunction. Thus, all analyses that included a variable of the PRE phase were performed using *n* = 9. Wilcoxon’s test was used to analyze the overall 2 × 2 design (session × exercise), and to compare related variables. Data gathered during the exercises was analyzed by a repeated measures ANOVA with exercise (2) as a within-subject factor and gender as a between-subject factor. The software used to perform the statistical analysis was the SPSS predictive analytics, version 23.0 (SPSS Inc., Chicago, IL, United States).

## Results

### Recovery Time

**Fig. 3:**
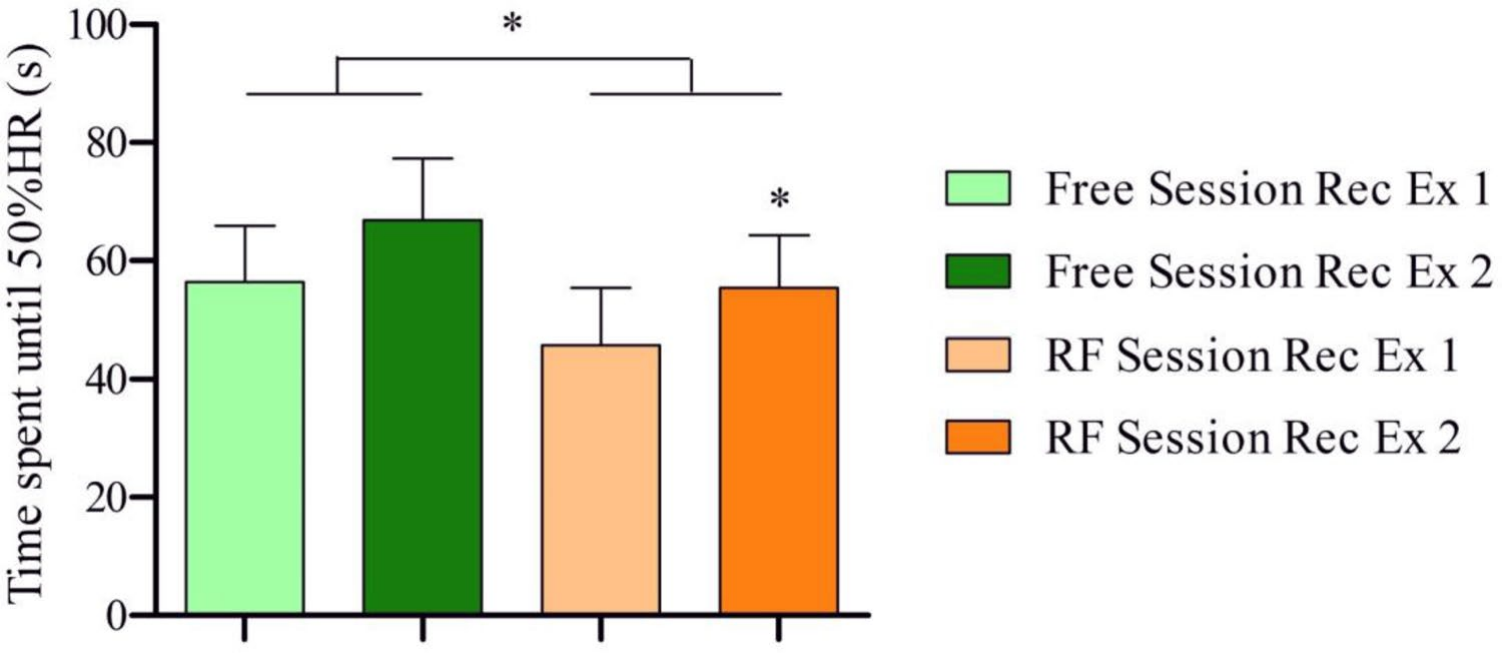
Time spent from the beginning of recovery until reaching 50%HR (Recovery time). *Rec* means recovery and *Ex* means exercise. Comparison between the mean of each session using *Wilcoxon’s test* (*RF* vs *Free*). Comparison between the corresponding recovery of the other session using *Wilcoxon’s test* (*RF session Rec Ex 1* vs *Free session Rec Ex 1*; *RF session Rec Ex 2* vs *Free session Rec Ex 2*). *p < 0.05. *n* = 10

As regards the recovery phase, the recovery time (time spent until 50% of HR) in the RF session was significantly lower than in the Free session (*Z* = − 2.073, *p* = 0.038). Furthermore, the recovery time in the first exercise of the RF session had a tendency to be significantly lower than in the first exercise of the Free Session (*Z* = − 1.897, *p* = 0.058). Similarly, recovery time in the second exercise of the RF session was significantly lower than in the second exercise of the Free Session (*Z* = − 1.956, *p* = 0.05) (see Table 2 and Fig. 3).

**Table 2:** Data gathered during the exercises

Variables	Free session		RF session	
	Exercise 1	Exercise 2	Exercise 1	Exercise 2
*Recovery*				
Time to 50% HR	56.40 (30.0)	66.90 (32.8)	45.80 (30.2)	55.40 (28.2)*
HR mean	94.30 (12.3)	99.40 (11.0)	88.50 (13.3)*	94.60 (12.3)*
RR mean	654.09 (86.1)	619.47 (70.4)	723.10 (110.3)**	669.42 (85.7)**
SDNN	63.21 (16.6)	62.67 (21.4)	115.57 (32.5)**	99.86 (32.4)**
RMSSD	35.39 (21.7)	24.57 (20.1)	65.87 (36.1)*	47.50 (26.5)**
LF	1311.69 (1134.9)	1835.30 (2410.7)	8305.09 (5297.1)**	5930.47 (5122.7)**
*Exercise* *performance*				
Time spent running	369.10 (127.2)	353.20 (127.3)	417.10 (151.4)**	385.30 (139.1)**
*Subjective measures*				
TQRper	6.90 (0.5)		7.80 (0.2)	
CR-10	3.30 (0.4)		4.80 (0.6)*	

Values are expressed as mean (SD); *n* = 10 (7 men and 3 women). *Time* expressed in seconds for *Time spent running* and *Time to (achieve) 50% HR*; values in milliseconds (ms) for *RR mean, SDNN* and *RMSSD*; values in ms^2^ for *LF*

*p < 0.05, **p < 0.01 vs the corresponding exercise of the free session

### HR and HRV Parameters During Recovery Phase

**Fig. 4:**
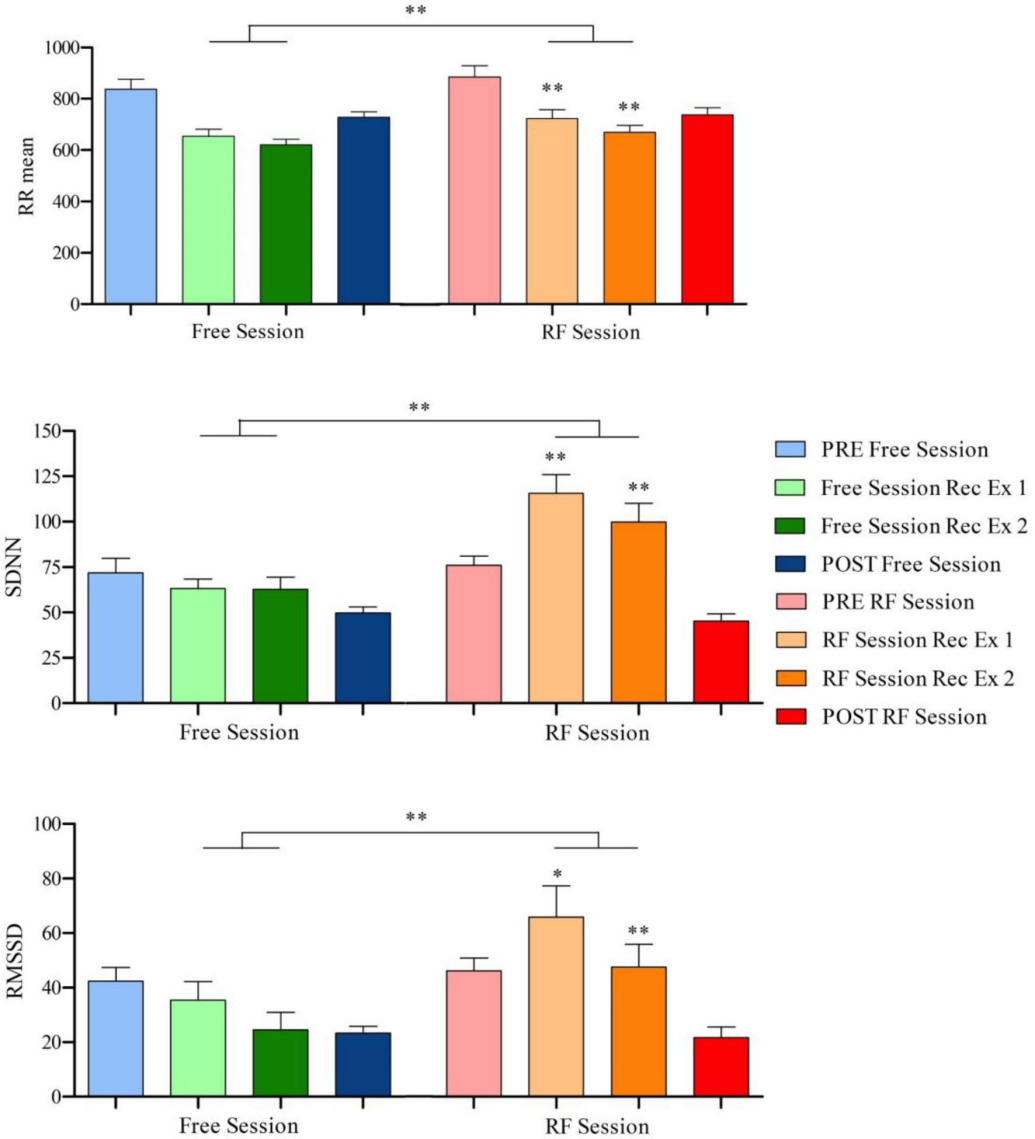
HRV parameters during PRE, recovery of exercises and POST. *Rec* means recovery and *Ex* means exercise. Comparison between the mean of each session using *Wilcoxon’s test* (*RF* vs *Free*). Comparison between the corresponding recovery of the other session using *Wilcoxon’s test* (*RF session Rec Ex 1* vs *Free session Rec Ex 1*; *RF session Rec Ex 2* vs *Free session Rec Ex 2*). *RR mean, SDNN and RMSSD* expressed in milliseconds. *p < 0.05, **p < 0.01. *n* = 10

**Fig. 5:**
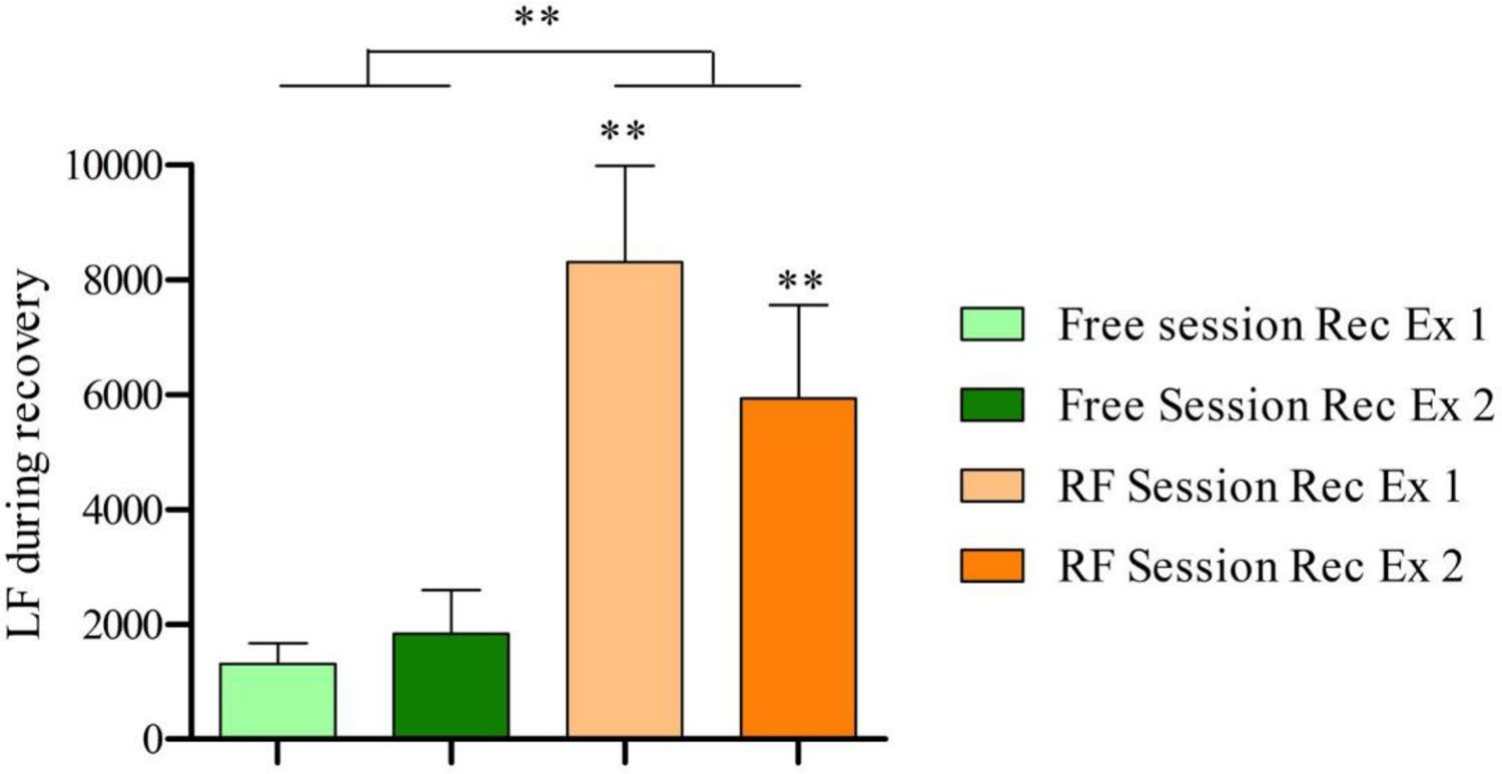
LF during recovery from exercises. *Rec* means recovery and *Ex* means exercise. Comparison between the mean of each session using *Wilcoxon’s test* (*RF* vs *Free*). Comparison between the corresponding recovery of the other session using *Wilcoxon’s test* (*RF session Rec Ex 1* vs *Free session Rec Ex 1*; *RF session Rec Ex 2* vs *Free session Rec Ex 2*). *LF* expressed in milliseconds squared (ms^2^). **p < 0.01. *n* = 10

Within the recovery phase (Table 2 and Fig. 4), the mean HR was significantly lower in the RF Session when compared to the Free Session (*Z* = − 2.346, *p* = 0.019). Moreover, in the RF Session the heart rate average (HRmean) in the first exercise and in the second exercise was significantly lower when compared to HRmean in the respective first exercise and second exercise of the Free Session (*Z* = − 2.176, *p* = 0.03; *Z* = − 2.321, *p* = 0.021). Regarding time domain HRV parameters, during the recovery phase of the RF session, higher values were obtained for RRmean (*Z* = − 2.803, *p* = 0.005), SDNN (*Z* = − 2.803, *p* = 0.005) and RMSSD (*Z* = − 2.803, *p* = 0.005) compared to recovery phase of the Free session. Furthermore, significantly higher values were obtained in the first exercise and second exercise of the RF Session compared to the values obtained in the respective first exercise and second exercise for RRmean (*Z* = − 2.803, *p* = 0.005; *Z* = − 2.803, *p* = 0.005), SDNN (*Z* = − 2.803, *p* = 0.005 and *Z* = − 2.701, *p* = 0.007) and RMSSD (*Z* = − 2.497, *p* = 0.013 and *Z* = − 2.599, *p* = 0.009). Regarding frequency domain parameters analysis, in the recovery phases, LF was significantly higher in the RF session when compared to the Free Session (see Fig. 5; *Z* = − 2.803, *p* = 0.005). Moreover, the LF in the first exercise and second exercise of the RF Session was higher when compared to LF in the respective first exercise and second exercise of the Free Session (*Z* = − 2.803, *p* = 0.005 and *Z* = − 2.803, *p* = 0.005).

### Exercise Performance

**Fig. 6:**
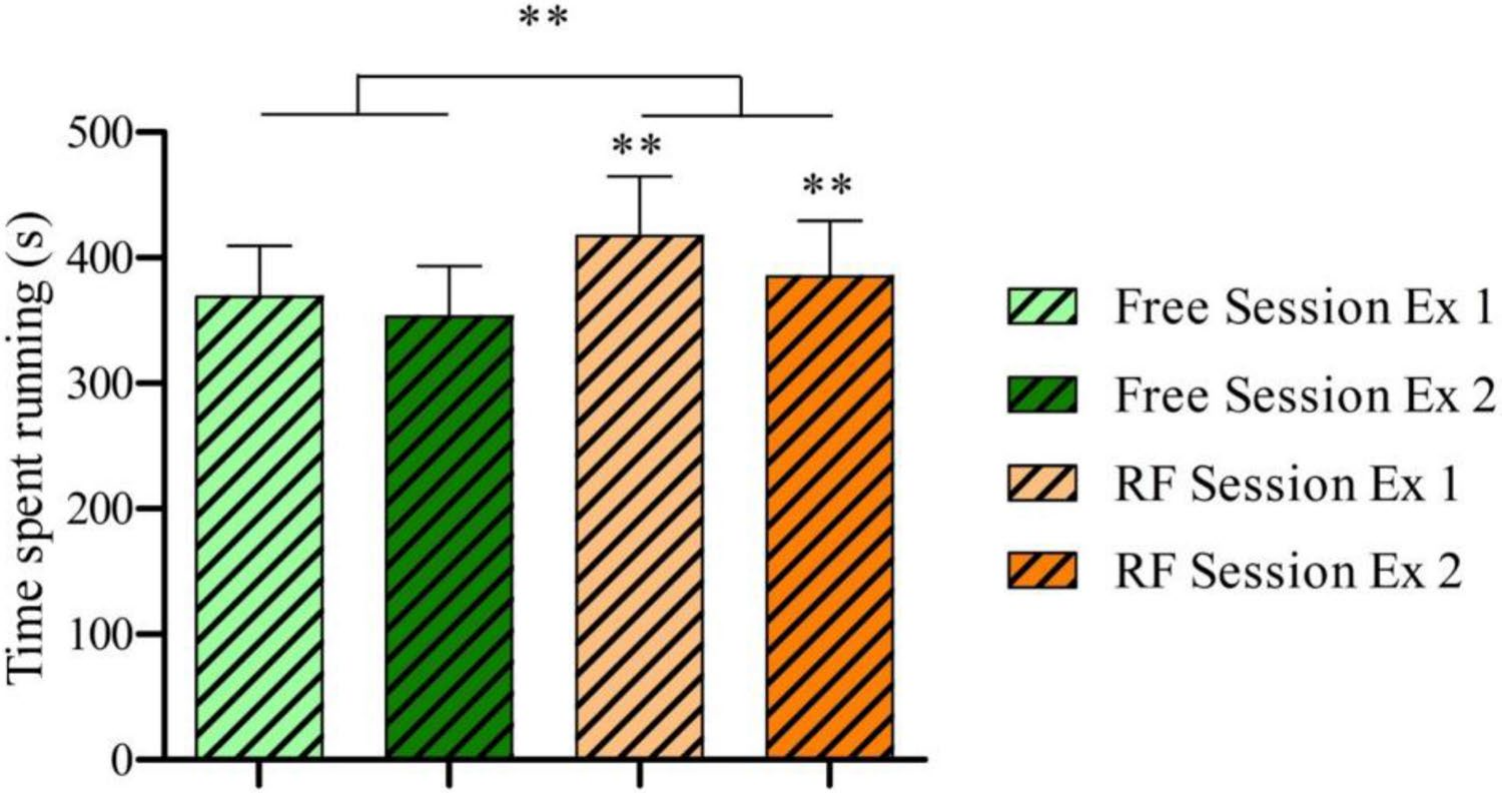
Time spent running from the beginning of the warm-up until reaching 80%HR. *Ex* means exercise. Comparison between the mean of each session using *Wilcoxon’s test* (*RF* vs *Free*). Comparison between the corresponding exercise of the other session using *Wilcoxon’s test* (*RF session Rec Ex 1* vs *Free session Rec Ex 1*; *RF session Rec Ex 2* vs *Free session Rec Ex 2*). **p < 0.01. *n* = 10

As regards exercise performance (see Table 2 and Fig. 6), the time spent running was significantly longer in the RF Session than in the Free Session (*Z* = − 2.666, *p* = 0.008). Furthermore, the time spent running during the first exercise and during the second exercise of the RF Session was significantly longer than the respective first exercise and second exercise of the Free Session (*Z* = − 2.666, *p* = 0.008; *Z* = − 2.668, *p* = 0.008).

### Subjective Measures

In the subjective measures (see Table 2), the TQRper scores in the RF session had a tendency to be significantly higher than in the Free Session (*Z* = − 1.710, *p* = 0.087). The CR-10 scores were significantly higher in the RF session than in the Free Session (Z = − 2.539, *p* = 0.011).

### Gender Comparison for the Effects of HRVB

The results show no gender differences by any HRV parameter as effects of HRVB intervention, comparing *Free* an *RF* sessions. There are also no gender differences in recovery and performance times.

### PRE and POST Heart Rate Variability Comparisons

No significant differences were found between the PREs and the POSTs of each session in any HRV parameters. Similarly, no significant differences were found in HRV parameters when comparing the mean of PRE and POST of RF session against the mean of PRE and POST of Free session (see Table 3).

**Table 3:** Data gathered before (PRE) and after (POST) sessions

Variables	Free session		RF session	
	PRE	POST	PRE	POST
HR mean	72.9 (10.0)	83.0 (7.4)	69.2 (10.7)	82.4 (9.7)
RR mean	836.9 (118.4)	728.1 (62.2)	884.4 (133.4)	736.7 (85.2)
SDNN	71.8 (23.8)	49.7 (9.8)	75.9 (15.1)	45.2 (11.9)
RMSSD	42.4 (15.0)	23.3 (7.7)	46.1 (14.2)	21.7 (11.8)
LF/HF	4.4 (3.6)	10.5 (10.3)	8.0 (13.6)	10.3 (10.5)

*Time* expressed in milliseconds for *RR mean.* Values are expressed as mean (SD). *n* = 9

## Discussion

This study was aimed at assessing the effects of HRV biofeedback (HRVB) on effort recovery. Participants underwent a 4-session procedure that started with the first session performing an aerobic exercise (running on a treadmill). Measurements included HRV parameters, running time, and recovery time, as well as subjective recovery and exertion. The following two sessions served to establish and practice HRVB. The final session was almost identical to the first one, with the single difference of applying HRVB during recovery. Results show improvement in all main HRV parameters, with lower recovery times and higher exertion.

From the moment that the participant sits down and starts recovering from a physical exertion, HR naturally starts falling (Peçanha et al. 2017), as the autonomous nervous system shifts from the predominantly sympathetic to the parasympathetic branch (Michael et al. 2017). The faster recovery shown in this study can be explained as a result of higher HR amplitudes produced by breathing at RF, which activates the PNS faster than in normal recovery. Breathing at RF makes HR rise and fall with more extreme peaks and valleys, making it more likely to achieve the 50% of the maximum HR faster (Lehrer 2013; Lehrer and Gevirtz 2014). Differences in SDNN show a clear effect on HRV when breathing at RF. Differences in RMSSD imply differences in vagal tone, indicating higher activation of the PNS (Shaffer and Ginsberg 2017). According to Thayer et al. (2009), higher vagal tone relates to better executive cognitive performance (key in sports), emotional, and health regulation. Furthermore, in view of the fact that vagal activity protects the cardiovascular system (Peçanha et al. 2013), prompt vagal recovery means protection against cardiac injuries for the athlete. Also, higher PNS activity correlates positively with recovery according to Chen et al. (2011). The improvement in recovery can be explained by the previously mentioned RF breathing effect that produces higher variability in the HR and it has two main effects. Firstly, aiding the cardiovascular system in adapting to the changes in exteroceptive and interoceptive demands (in this case from a stressful environment to a non-stressful environment): thus returning to a faster and better adjusted rest (Lehrer and Eddie 2013). Secondly, increasing efficiency in gas exchange that takes place in the alveoli (Hayno et al. 1996; Yasuma and Hayano 2004; Ramos-Campo et al. 2017), therefore providing an enhancement in the oxygen uptake and the reduction of blood carbon dioxide levels, characteristic of the process called Excess Post-Exercise Oxygen Consumption (EPOC; LaForgia et al. 2006).

Participants were able to correctly assess their internal load, evidenced by the fact that both perceived exertion and the time-measured exercise performance increased (Borresen and Lambert 2008). Although exercise performance and perceived exertion of that session was significantly higher, the perception of recovery after the RF session was close to significance when compared to the Free session. This meant that the sample of participants, thanks to the HRVB, had a tendency to perceive that they were more recovered. This impact on a subjective measurement suggests that more psychological measures related to recovery should be taken into consideration when assessing the quantitative and qualitative consequences of HRVB on recovery, such as the Acute Recovery and Stress Scale and the Short Recovery and Stress Scale (Nässi et al. 2017).

As regards the comparison of PRE and POST measurements between Free and RF session, the most interesting result is the absence of significant differences between the PREs of both sessions. This result is indicative of similar levels of physical fitness (Buchheit 2014) and stress (Morales et al. 2013), meaning that the participants arrived at both sessions in a similar state. Moreover, this result shows that there is no long-lasting or chronic effects from the RF detection sessions that could have improved HRV parameters, therefore disregarding common persistency effects from HRVB training (Deschodt-Arsac et al. 2018) and implying only acute effects on HRV.

The design of the procedure was robust because of the differences seen in both SDNN and RMSSD. While both are parasympathetically influenced, SDNN is more reliable against artifacts (because abnormal beats have been removed); and even though RMSSD is more susceptible to artifacts, it correlates highly with vagal tone (Shaffer and Ginsberg 2017). Moreover, the signal error average for all RR records was 0.67%, therefore measurements of both parameters are valid.

Differences in LF showed that the participants were breathing at a low frequency within the recovery period, as they were supposed to (see Fig. 5). LF is composed by frequencies that lie between 0.04 and 0.15 Hz, and the participants were instructed to breath in frequencies from within that range (0.08–0.12 Hz; Laborde et al. 2017). This meant that if the value of LF was higher in the session in which participants had to breath at a low frequency (RF session) in comparison to the session in which they had no instruction (Free session), then they were indeed breathing within the range that they were supposed to. This effect is due to RSA (Steffen et al. 2017).

Vaschillo et al. (2006) find gender differences in HRVB, but we did not find any gender difference in the effects of HRVB. Possibly in our case it is due to the small size of the sample (there are only 3 women and 7 men in the final sample). Our goal was not gender comparison, but the normal differences in running performance between women and men could affect the results. Therefore, it would be interesting to analyze possible gender differences in HRVB with a sufficiently large sample and balancing it according to the level of physical activity.

The main limitation of the study was the difference shown in exercise performance between the first exercises of each session. The first exercises of each session should not have resulted in significant differences, because up to these instances both sessions were equal. Only after the first exercise, Free session and RF session were different: the first had no breathing instruction and the latter did. This effect has different explanations. Improvements in fitness in some participants could have been noticeable in the time spent running, but not in the HRV parameters during PRE assessment (as indicated above). This mismatch has been reported in previous literature, and the fitness-related link between distance-based performance and HRV parameters has still to be clarified (Buchheit 2014). Another explanation could be that the participants got used to running on the treadmill. Fixing this limitation could be done by adding a specific session to accustom participants to the instruments and experimental context, or comparing participants to a control group, or adding a second RF session at the end, or using an incremental maximal running test (Weberruss et al. 2018). The latter suggestion would be rather invasive, and we did not want to change the daily routine of the participants. The use of a control group is not the best option, because of difficulties in interpersonal comparisons of HRV (Laborde et al. 2017).

There are several applications suggested by the results of this study. Every time an athlete performs, they exert a physical and psychological effort that needs to be recovered from; a process that takes time and can be improved by following these specific instructions. To the best of our knowledge, HRVB has never been used before during short-term recovery. The effective decrease in recovery times and the improvement in the quality of such recovery would allow the athletes to return to competition faster and in a better psychophysiological state. Sports with intermittent loads such as basketball, handball, volleyball, tennis, etc., would particularly benefit from this, because of their characteristic exertion-pause-exertion nature that provides intervals to apply HRVB. During competition, many periods of time are available for applying HRVB, such as during substitutions, in between points, games and sets, during halftime, etc. (Lagos et al. 2011; Shaw et al. 2012). Emotional regulation is another benefit that the application of HRVB can bring, such as demonstrated in a study by Gross et al. (2016) using similar principles. Recovery after exercises in training sessions would also be shorter (therefore freeing training time) and prepare the athlete better for the next activity. Amateur athletes could benefit from HRVB too, for example by using it after an aerobic bout of exercise.

Further research can provide a wider and deeper knowledge of the application of HRVB during recovery. First of all, improving the HRVB protocol by making the participants breathe longer at RF should provide more noticeable effects and even benefits. Using different physical tests, such as an incremental maximal running test, could check the HRVB effectiveness in different situations. Conducting research on a sport-specific sample would shed light on distinguishing appropriate sport settings that would benefit from using this HRVB protocol and its effects on sport performance. Applying it on physically non-active participants may corroborate assumptions on whether HRVB works in such population. Assessing more psychological variables, such as relaxation (a stress management protocol with similar theoretical foundations have proven effective, Gross et al. 2018), concentration, emotion regulation, anxiety, and mood, could show advantages not previously thought of. Enlarging the sample may give rise to increasing differences that, in the current sample, were not large enough to result in significance. It would be beneficial to try to integrate our HRVB protocol to competition and training environments, in order to assess specific effects and obstacles. HRVB should also be applied in early beginners. It is important that young athletes become aware of the availability of such a beneficial technique that bring improvements in wellbeing and performance. Finally, researching the effects of the HRVB protocol on sport-related outcomes (such as scoring, passes completed, shots taken, etc.) would be of great interest.

## Conclusion

Our results show that using HRVB during recovery improves cardiac variability and the time spent exercising, lowers recovery time, and improves the perceived physical exertion and the recovery perception. Faster and better recovery is an advantageous resource in current competitive sport environments. The benefit–cost ratio of applying HRVB during recovery would make it highly valuable to a handful of sport-related situations. The rapid establishment of personal RF and the effortless change in the inherent physiological process of breathing, give rise to powerful effects on recovery and, therefore, to the practice of sport itself.
